# A brief history and review of modern casting techniques in early onset scoliosis

**DOI:** 10.1007/s11832-016-0762-4

**Published:** 2016-07-28

**Authors:** Ozgur Dede, Peter F. Sturm

**Affiliations:** 1 Department of Orthopaedic Surgery, Children’s Hospital of Pittsburgh of University of Pittsburgh Medical Center, 4401 Penn Avenue, Faculty Pavilion 4th Floor, Pittsburgh, PA 15224 USA; 2 Department of Orthopaedic Surgery, Cincinnati Children’s Hospital Medical Center, 3333 Burnett Avenue, MLC 2017, Cincinnati, OH 45229 USA

**Keywords:** Body cast, Early onset scoliosis, Mehta cast, Risser cast

## Abstract

**Purpose:**

Body casts have a long history in the treatment of spinal deformity. Currently the use of body casts is limited to early onset scoliosis. Here, we aim to provide a brief narrative of the evolution of cast application for the management of spinal deformity.

**Methods:**

A history of cast application is provided with a brief review of the orthopedic literature. The current indications for cast application and the authors’ preferred technique are described.

**Results:**

Serial casting is an effective treatment method for early onset scoliosis. It may be definitive for most idiopathic curves or used to delay surgical intervention in more severe idiopathic, syndromic and even congenital curves.

**Conclusions:**

Surgeons who treat children with early onset scoliosis should familiarize themselves with serial cast application techniques.

## Introduction

Today, the use of plaster casts in the treatment of scoliosis is almost completely confined to the management of early onset scoliosis (EOS). The use of an external support device to correct scoliosis dates back to Ambroise Paré [1]. The application of plaster casts, on the other hand, is a relatively new method; the popular use of plaster of Paris (POP) began as recently as the 1800s [2]. Here, we aim to examine the evolution of cast application in the treatment of EOS and present the authors’ current indications and technique.

## The history of cast treatment in scoliosis

Even though the commonplace use and acceptance of serial body casts for the treatment of EOS is recent, plaster cast use in the treatment of spine deformity dates back to the 19th century. After the introduction of POP, it did not take long until POP was adapted to spinal deformity treatment. Lewis Sayre reported his technique and experience on how to correct spinal deformities with traction (partial suspension) and application of POP cast in 1877 [3]. Sayre’s book ‘Spinal Disease and Spinal Curvature: Their Treatment by Suspension and the Use of the Plaster of Paris Bandage’ consisted of two parts—angular kyphosis and rotary lateral curvature of the spine. The 10 patients who were described in the section on rotary lateral curvature of the spine were mostly adolescent females who appeared to have moderate-sized idiopathic curves. Bradford and Brackett presented a technique of lateral pressure and cast application in 1893 [4]; their report begins with “The method of treatment of resistant lateral curvature of the spine by means of mechanical correction is not new. It has until recently, however, fallen somewhat into disfavor, superseded largely by gymnastic methods”. This exemplifies another swing of the pendulum between different treatment methods in the world of orthopedic surgery as that same sentence could have been used at the beginning of the present review. Bradford and Brackett presented five cases along with a detailed description of their traction device and cast application method. One of their cases was a 4-year-old child and a straight spine was achieved after one year of cast treatment.

After the surgical fusion of spinal curves was introduced, the use of plaster casts was better defined by Hibbs as “a corrective cast was used to achieve a preliminary correction before surgical fusion and then postoperatively to achieve additional correction and hold the correction until the bony fusion was achieved” [5]. Risser provided a more detailed description on how to apply a corrective cast in 1955 [6]. He described the use of a specialized table during cast application for curve correction. The patient lay supine on this special table frame supported by a longitudinal hammock strap with horizontal supports under the pelvis/thighs and shoulders. Longitudinal traction was applied via pelvic straps and a halter-type occipito-mental strap. A ‘localizer’ was used to apply focused corrective force in order to achieve a better correction as the cast was applied. The localizer consisted of a metal arch that could be screwed up to apply posterolateral pressure on the rib prominence to correct the rotation and the lateral curvature. A window was cut out in the back of the cast and the surgical fusion was performed through this window. After the surgery, the cast was changed after approximately 7–10 days and then every 3–4 months until solid fusion was achieved [7]. Risser hoped that the localizer body cast could be utilized to correct the scoliosis and obviate surgical treatment in the very young; however, no results for this technique were reported [8]. After the advent of Harrington instrumentation, the need for external immobilization decreased [9]. Later, with the development of segmental instrumentation, postoperative cast support became obsolete [10]. While the use of plaster cast application slowly faded from the treatment of juvenile and adolescent patients in favor of braces and surgery, serial cast application was found to be effective in correcting idiopathic early onset spinal deformities [11].

A report by Cotrel and Morel is the first strong suggestion that serial casting might be used as a corrective measure in EOS [11]. In their report, they detailed the casting technique and presented their results in 75 patients with idiopathic scoliosis. Cotrel and Morel’s technique differs from Risser’s method in the way that de-rotational force is applied. Both traction and posterolaterally directed force over the rib prominence was used in Risser’s localizer cast method which implies that Risser also employed de-rotation of the thoracic cage and therefore the spine. On the other hand, Cotrel and Morel applied the corrective force through straps instead of a localizer and specifically called it de-rotation. In the description of their elongation, de-rotation and lateral flexion (EDF) method they specified the corrective forces on the spinal column—elongation is achieved by applying longitudinal traction to the spine through pelvic straps and halter head traction. De-rotation is achieved with straps that wrap around the rib prominence and pull in an oblique fashion perpendicular to the ribs. Lateral flexion is also achieved by straps pulling laterally as well by adjusting the pelvic and halter traction in a differential manner. Although the results were not presented in detail, the authors indicated that if the treatment is started early in infants with scoliosis, spinal growth may be harnessed in order to control the deformity progression. In juvenile cases they used the cast application as an adjunct to posterior arthrodesis, similar to the turnbuckle and localizer body casts.

Although the above-mentioned authors implied or suggested the use of cast application as a treatment method in early onset curves without arthrodesis, the first formal report of serial casting specifically addressing idiopathic EOS was presented by Mehta and Cotrel at the ‘Sixth Symposium on Scoliosis’ held at the Cardiothoracic Institute in London in 1979 [12] (now known as the International Phillip Zorab Symposium). In a previous report, Mehta had shown that EOS could be classified into resolving and progressive types and that these types could be differentiated using the rib vertebral angle difference and the rib stage [13]. Mehta further delineated the progressive type as being either benign progressive or malignant progressive subtypes. Their report consisted of 21 children with progressive benign infantile scoliosis who received early body cast application [12]. At the time of their report, six of the patients were skeletally mature with curves maintained at ≤21 degrees. The remaining patients, who were still growing, also had well-controlled curves. Although they did not detail the cast application technique, they noted that thoracic and thoracolumbar curves correct better with lateral bending or wedge-type casts, and that thoracic and lumbar combined curves correct better with distraction-type casts.

Twenty-five years later, Mehta published her prospective study on the cast treatment of infantile progressive scoliosis on 136 patients [14]. The cast application was performed following the method of Cotrel and Morel; however, Mehta did not use straps for de-rotation but instead the cast was molded with manual pressure over the rib prominence. The spinal deformity was completely corrected if the treatment with casting started early in children with moderate curves. Cast treatment did not resolve the curves in older children with severe curves; however, the curve magnitude was effectively reduced. Following this impressive prospective report, serial casting quickly became an accepted method for the management of EOS as both a definitive treatment and to delay surgical procedures. Detailed cast application techniques have been published previously [15–17].

### The rationale and outcomes of casting in EOS

A body cast for scoliosis provides a non-removable and well-molded jacket thus imparting a consistent corrective force on the young spine. In a small and fast growing child the frequent need for adjustments to accommodate the child’s growth makes brace use impractical. Although cast changes every 2–3 months do allow for these adjustments to the child’s growth, it does add the disadvantage of frequent general anesthesia. The fastest spinal growth occurs during the first year of life. Growth continues in the second year with a rate similar to the adolescent growth spurt. The quick growth pace continues until the end of the fifth year and slows down between the fifth and tenth years [18, 19]. Serial casting aims to utilize this fast growth as the corrective force. During cast application, axial traction is applied in order to ‘elongate’ and de-rotate the spinal deformity; additional de-rotation is achieved by manual manipulation over the rib prominence and the corrected position is then held by the cast. In her practice-changing report, Mehta [14] explained that the shape of an organ or part determines the direction of its continued growth “as long as the direction remains constant, growth will simply perpetuate and enlarge its existing shape”, i.e., if a curved spine is left untreated, it will grow more curved. The theory is that with the external force of the cast, especially during a period of rapid growth, the spine will continue to grow in the corrected position and the final shape can be altered.

Success of cast treatment is correlated with treatment beginning during the first two years of life. In the study by Mehta [14], all curves in patients (with syndromic or idiopathic scoliosis) who began casting between 15 and 21 months of age with Cobb angles between 27° and 35° resolved; conversely, curves in patients who began their treatment between 27 and 34 months of age with Cobb angles between 47° and 53° did not resolve. Further evidence has shown that age at initiation of treatment is a major factor; idiopathic curves up to 60° can resolve if treatment begins before the age of 2 years [15].

For those patients whose curves cannot be definitively treated with serial casting, the treatment can still serve as a means to delay surgical intervention and its associated complications including wound healing problems, infection, spontaneous fusion, implant failure and many others [20–22]. In a study of serial casting outcomes conducted by Fletcher et al. [23], surgery was delayed in 15 of 29 patients by 39 ± 25 months (the equivalent of nearly seven growing rod lengthenings) from the time of their first cast. The remaining 14 patients had not required surgery at publication. Overall, 72 % of the patients had avoided surgery at an average follow-up of of 5.5 years. These patients had both congenital and neuromuscular diagnoses with Cobb angles >50° and did not begin their casting treatment until an average age of 4.4 years. The good results of cast treatment are not exclusive to idiopathic scoliosis. Waldron et al. [24] reported the results of the use of serial cast application in 20 children with EOS secondary to mixed etiologies. At the time of the report, five children were being braced, six were still undergoing cast changes, seven underwent growing rod surgery and two were lost to follow-up. In cases where there was continuous curve progression, the need for additional intervention was delayed for an average of 16.8 months.

Enthusiasm for cast treatment has been increasing as clinical reports become available. A recent study comparing growing rod application versus cast treatment in age- and curve-matched cohorts showed that cast treatment is a viable option for children with EOS and avoids the complications associated with growing rod instrumentation [25]. The authors compared two cohorts of 27 patients, one group was treated with growing rod instrumentation and the other group was treated with serial casting. Although the growing rod group achieved better curve correction, there were 23 complications. In the cast group, however, the magnitude of deformity was maintained rather than decreased, but significantly there was only one skin complication. The study included children who were older than the age group in which cast treatment is most successful; however, the ability to prevent progression is very important as it delays surgical intervention. It is known that with every year of growing rod treatment the rate of complications increases by approximately 24 % [26].

### The role of serial casting in congenital scoliosis

Traditionally, cast treatment has been regarded as futile in congenital scoliosis, but occasionally used to control the compensatory curves [27, 28]. A recent report by Demirkiran et al. challenges this opinion and suggests that even congenital curves may benefit from serial casting in order to delay surgical interventions [29]. The patient cohort of their study included 11 patients with an average age of 39.5 months at the time of initial cast application. All curves consisted of long anomalous segments with at least five abnormal vertebrae. At the time of last follow-up the congenital curves were decreased from an average of 70°–55° and the compensatory curves were decreased from an average of 56° to 40°. The sagittal deformity was also corrected in the cast from an average of 76° to 64°. As the final curve magnitudes were in cast measurements, the authors did not claim to have achieved correction; however, at the last follow-up serial casting did delay the need for surgical intervention for an average of 26 months while preserving growth of the spine. At the time of the last follow-up, nine patients were still undergoing serial cast changes and two patients underwent growing rod surgery.

### Problems associated with serial cast treatment

Serial casting prevents most complications associated with spinal fusion or growing rod surgery but it can present a different set of problems. The most common problems are minor skin complications that do not require formal treatment. On the other hand, a case report of lateral subclavian vein thrombosis in a patient with a Cotrel cast proves that serial body cast treatment is not free of more severe complications [30]. This serious potential complication may be avoided by careful and generous trimming of the cast at the groin and axillary regions. Another valid concern is that the application of a rigid and molded cast over the chest wall may impair ventilation. A recent study investigating the change in pulmonary pressures during and after the application of scoliosis casts in children with EOS, reported increased peak inspiratory pressures during the application of the cast [31]. After the application of the cast, the average peak inspiratory pressure increased by 106 % when compared with the baseline value. Following the removal of the thoracoabdominal cut-out, the average pressure decreased; however, it was still 32 % higher than the baseline value. Reported clinically significant problems were difficulty in maintaining ventilation during two procedures, one hypotensive episode, one case of hypoxemia after casting application, and breathing difficulties in one patient. We recommend routine intubation during cast application.

### The role of bracing during cast treatment

Bracing is often used as an adjunct to serial casting, but there is insufficient evidence to support the use of bracing as a sole management method. In a study comparing the different treatment methods for EOS, Smith et al. [21] showed that bracing was the only treatment that did not provide adequate curve control at their institution. Additionally, the authors noted that the patients for whom bracing treatment was effective had spontaneously resolving curves that may have improved without bracing due to their significantly smaller rib vertebra angle difference and Cobb measurements. Brace wear compliance is another major concern. There are no reports evaluating brace compliance in EOS patients. Compliance has been shown to be a challenge in other patient groups. Currently, bracing is used as an adjunct to cast treatment. It is typically used after satisfactory correction is achieved with casting or can be used during ‘cast holidays’ when patients are left cast free for a few months after one year of casting, e.g., during the summer season.

### The authors’ casting technique and treatment protocol for EOS

The cast treatment is started as soon as the progressive nature of the curve has been shown. We use the rib vertebral angle difference, rib stage and also the progression of the curve during follow-up visits as the criteria to start cast treatment versus observation.

We routinely apply the body casts under general endotracheal anesthesia. Following endotracheal intubation, a bite block is placed to protect the tongue and cheeks, as well as the tube, from accidental bite as traction is applied with a halter strap. A cast t-shirt is placed on the patient and two stacked towels are placed under the t-shirt to prevent the cast from being too tight. A stockinet may be used instead of the cast t-shirt. A towel is wrapped around the patient’s occiput and covers the ears as well as the eyes. This towel helps protect the ears from the pressure of the halter strap and is also useful to protect the face from the saw dust at the end of the procedure. Then, a halter strap is placed around the occiput and under the chin. Long straps are cut from a cloth wrap and placed around the patient’s waist above the iliac wings on both sides. These straps are placed over the cast t-shirt to prevent skin irritation during removal. The patient is then transferred onto the cast frame. Different cast frames are available commercially depending on the surgeon’s preference.

The back support of the frame is removed while the patient’s head is supported by the anesthesiologist and the torso and the legs are supported by the assistants. A horizontal bar is placed under the sacrum and another horizontal bar under the ankles to support the patient’s weight. The head is supported by a head rest and also with the traction applied through the halter strap. We routinely pad all supporting structures in order to prevent pressure-related problems. The arms are secured to the sides of the frame with cotton padding.

**Fig. 1:**
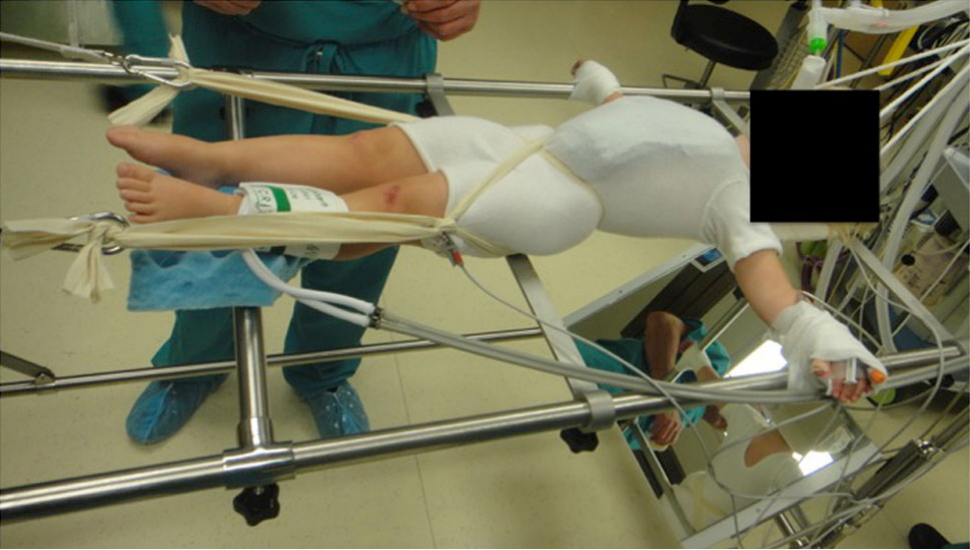
At the end of positioning, the patient is supported by the horizontal bar under the sacrum and the head support and partially suspended with the traction. Note the position of the flip mirror

The halter strap is connected to the ratchet mechanism at the head of the table and the pelvic straps are connected to the ratchet mechanisms at the foot of the table. Gentle traction is applied by gradually tightening the ratchets at the foot of the table. More traction is applied on the pelvic strap that is over the iliac crest of the concave side of the curve. At this point the surgeon should be aware of the amount of traction applied, as too much traction must be avoided. At this point the patient is supported by the horizontal bar under the sacrum and the head support and partially suspended with the traction (Fig. 1).

**Fig. 2:**
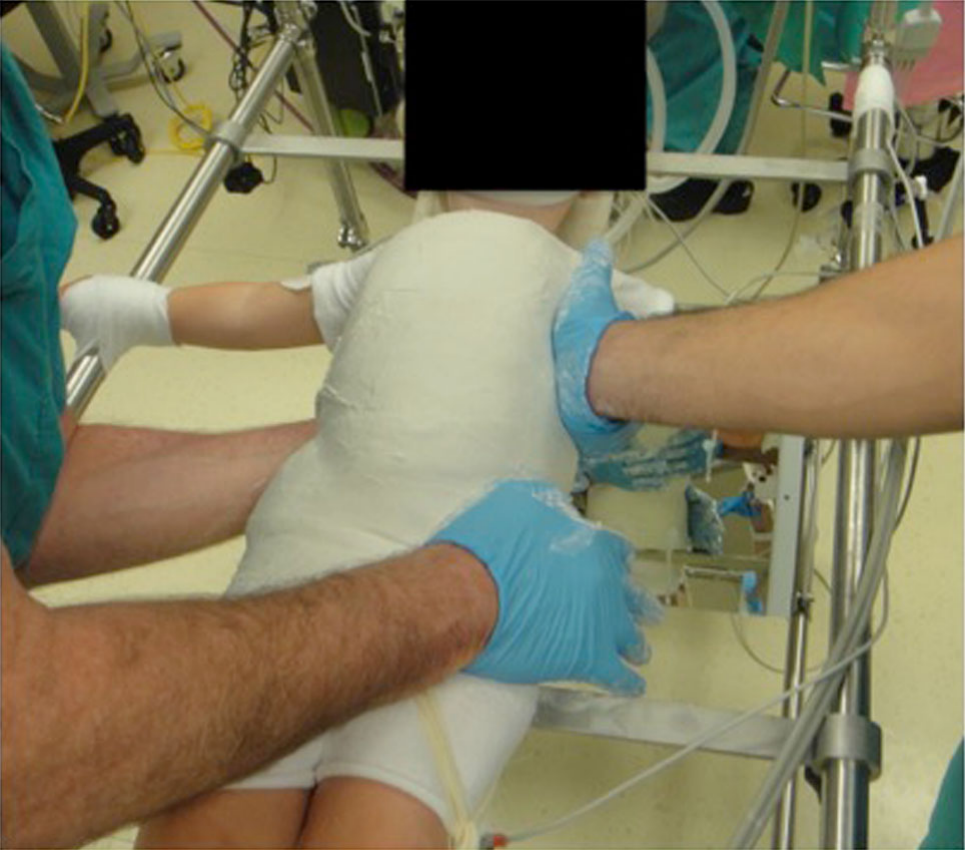
The surgeon (*left*) places one hand for the corrective mold at the posterolateral aspect of the convexity (over the apical ribs), and supports the pelvis contralaterally with the other hand. The assistant (*right*) applies counter pressure on the concavity of the curve

**Fig. 3:**
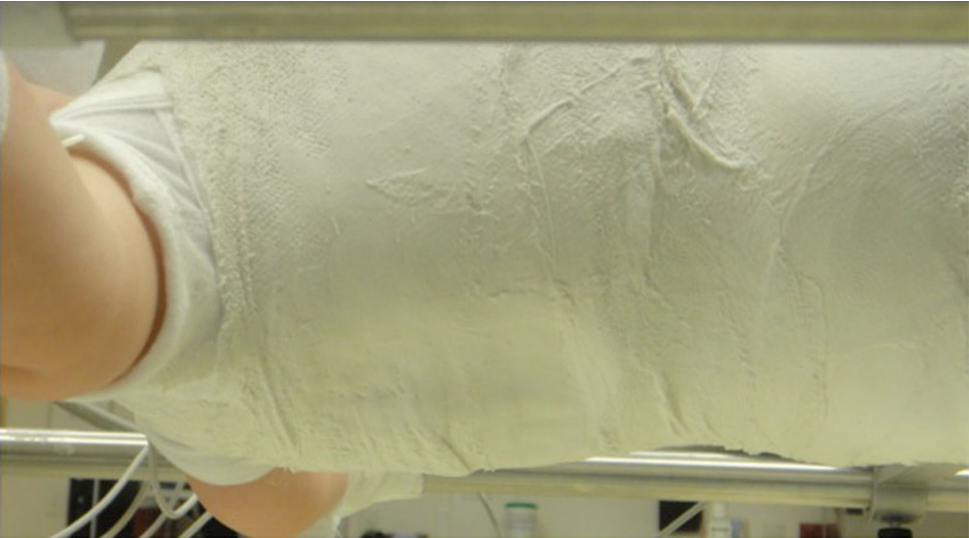
The mold on the convexity of the curve should be visible and palpable

Four to five layers of cotton padding are applied and padding is also placed where the shoulder straps will be placed (if used). Two circular felt pieces are placed over the anterior superior iliac crests and over-rolled with padding. Four layers of plaster of Paris are then rolled. During this process, approximately 2–3 cm wide plaster straps are fashioned as shoulder straps and incorporated into the cast. While placing the shoulder straps make sure that the assistants pull down on the straps until they are incorporated into the rest of the cast otherwise the straps will ride high. The shoulder straps are surgeon preference and one of the authors does not use the straps. The pelvic mold is the foundation of the cast and the iliac wings should be well molded. The surgeon then applies pressure with the palm of one hand to the prominence on the ribs that attach to the apex of the thoracic curve in a posterior to anterior and lateral to medial direction. The placement of this mold should be checked using the flip-mirror on the table. To counteract this pressure, the surgeon places his/her other hand on the contralateral iliac wing. The assistant places one hand proximally on the anterolateral aspect of the thorax as the third pressure point (Fig. 2). There should be a visible mold at the posterolateral aspect of the convexity (Fig. 3). Once the POP is set, fiberglass is applied for additional strength.

**Fig. 4:**
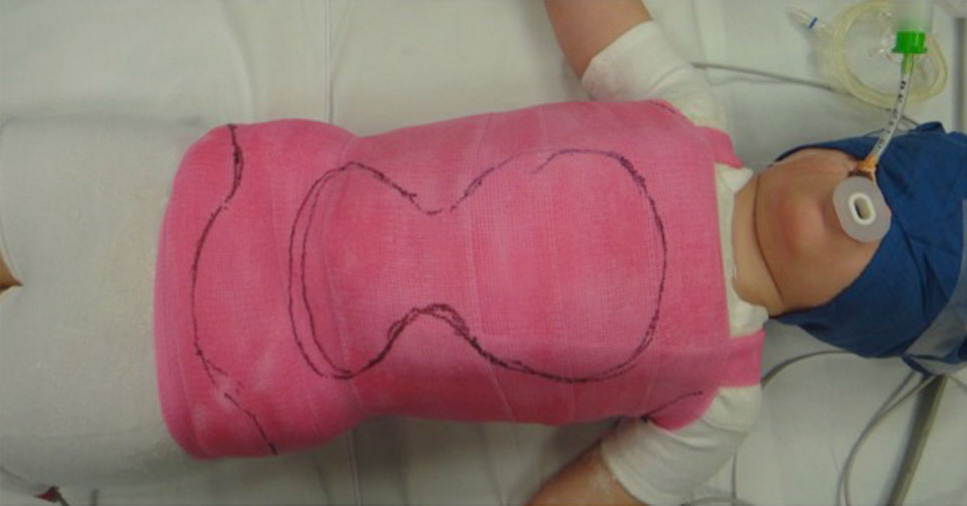
An anterior mushroom-shape cut-out is fashioned to allow for comfortable breathing and feeding while the side flares help prevent rotation of the rib cage

**Fig. 5:**
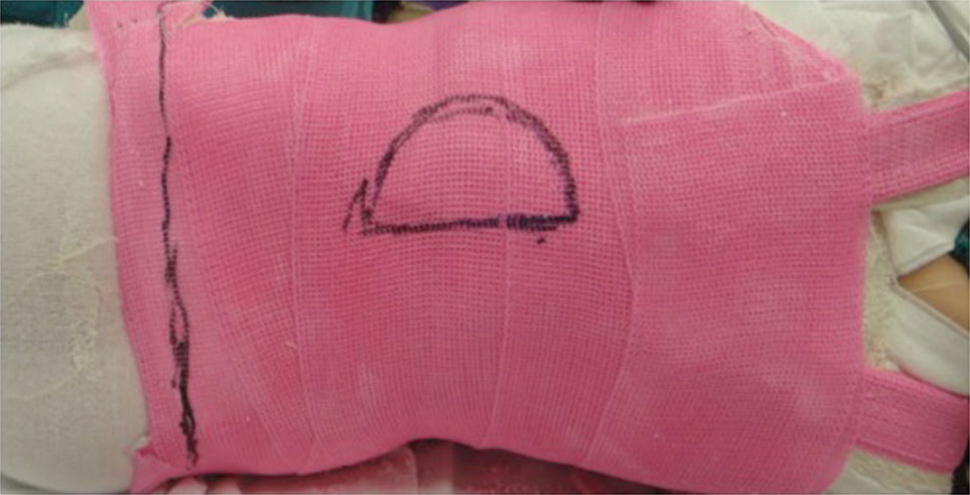
A posterolateral window is cut out on the concavity of the curve to allow for further de-rotation

Once the cast is set, the patient is transferred to the stretcher and an anteroposterior radiograph is obtained. The aim is for a correction of ≥50 % in the cast; however, this is not always possible. Once the radiograph shows satisfactory correction, the thoracoabdominal window is cut out in a mushroom shape to allow for decompression of the abdomen/diaphragm and also prevent the rib flares from rotating (Fig. 4). A posterolateral window is cut out on the concavity of the curve over the apical ribs to allow for further de-rotation (Fig. 5). The towels are removed, the cast is trimmed and edges are padded with moleskin. The skirt of the cast is trimmed to allow for 90° of hip flexion anteriorly and also posteriorly to allow for hygiene and care. The underarms are cut out wide enough to allow for unhindered shoulder motion. Proximal trimming may be necessary to prevent impingement of the chin on the chest portion of the cast.

Postoperatively, the patient is discharged unless there are other medical conditions prohibiting an outpatient procedure. The cast is changed every 2 months for children aged ≤2 years and every 3 months for children aged ≥3 years.

For children who achieve complete correction no further treatment is necessary. In children where complete correction is not achieved a brace is used to maintain the correction. There are no standardized indications on when to stop treatment or switch to bracing and most surgeons have their own preferences. If there is progression, serial casting is resumed. In progressive curves we aim to delay the surgical treatment (growing rod instrumentation) until the child is at least 5 years of age.

## Conclusions

Serial casting is an effective treatment method for EOS. It may be definitive for most idiopathic curves or as a means to delay surgical intervention in more severe idiopathic, syndromic and even congenital curves. Surgeons who treat children with EOS should familiarize themselves with serial cast application techniques.
